# Supplementary material for: Identification of photoperiod-induced specific miRNAs in the adrenal glands of Sunite sheep (Ovis aries)
Source: Front Vet Sci. 2022 Jul 22;9:888207. doi: 10.3389/fvets.2022.888207 (PMC9354845; doi:10.3389/fvets.2022.888207)
Supplement: Supplementary file 1 [file Data_Sheet_1.ZIP › Supplementary file/miRDeep2 file.zip!/miRDeep2 file/Novel_Structure/SPLP42A3/Novel_219.pdf]

Provisional ID	:	NC_019467.2_18931
Score total	:	1.6
Score for star read(s)	:	-1.3
Score for read counts	:	0
Score for mfe	:	1.3
Score for randfold	:	1.6
Score for cons. seed	:	
Total read count	:	8525
Mature read count	:	8525
Loop read count	:	0
Star read count	:	0

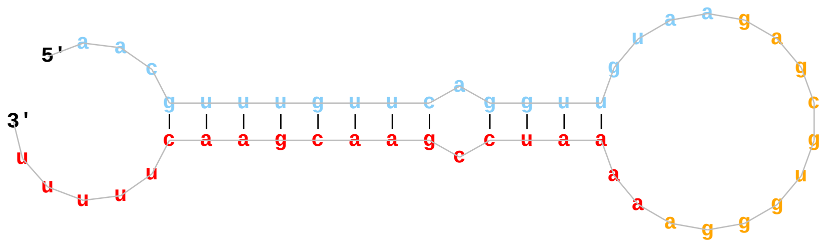

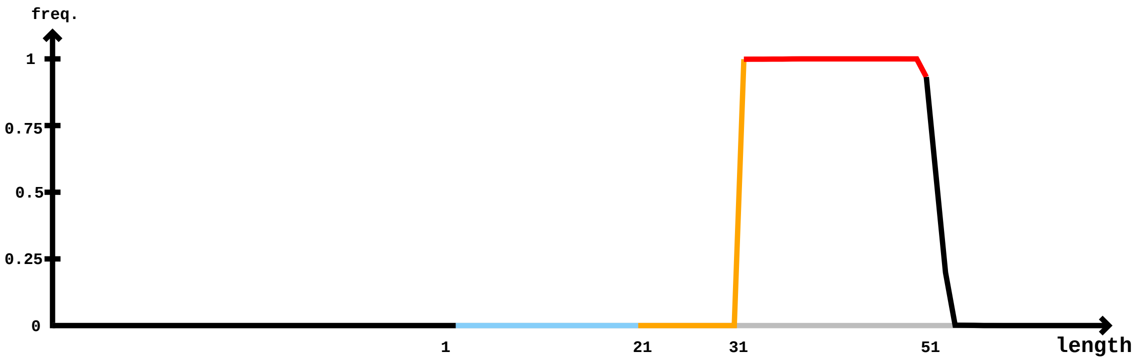

Star Mature

```
5'- uggguucuguccuagguaaugguaguacuggguuggccaaaacguuuguucagguuguaagagcgugggaaaaauccgaacgaacuuuuuggccaagucaauaauuug   -3'    exp
    ..(((.(((.((........))))).)))((.((((((((((.((((((((.((((...............)))).)))))))).)))))))))).)).........   reads      mm         sample
    ......................................................................aaaauccgaacgaacuu.....................   1          0          seq
    ......................................................................aaaauccgaacgaacuuuu...................   579        0          seq
    ......................................................................aaaauccgaacgaacuuuuu..................   6243       0          seq
    ......................................................................aaaauccgaacAaacuuuuu..................   6          1          seq
    ......................................................................aaaauccgaacAaacuuuuug.................   1          1          seq
    ......................................................................aaaauccgaacgaacuuuuugg................   1683       0          seq
    ......................................................................aaaauccAaacgaacuuuuugg................   2          1          seq
    .......................................................................aaauccgaacAaacuuuuuggc...............   2          1          seq
    .........................................................................auccgaacAaacuuuuuggcc..............   2          1          seq
    ...........................................................................ccgaacgaacuuuuuggcca.............   4          0          seq
    ...........................................................................ccgaacgaacuuuuuggccaag...........   1          0          seq
    ............................................................................cgaacgaacuuuuuggcca.............   2          0          seq
```
